# RiboToolkit: an integrated platform for analysis and annotation of ribosome profiling data to decode mRNA translation at codon resolution

**DOI:** 10.1093/nar/gkaa395

**Published:** 2020-05-19

**Authors:** Qi Liu, Tanya Shvarts, Piotr Sliz, Richard I Gregory

**Affiliations:** Stem Cell Program, Division of Hematology/Oncology, Boston Children's Hospital, Boston, MA 02115, USA; Department of Biological Chemistry and Molecular Pharmacology, Harvard Medical School, Boston, MA 02115, USA; Computational Health Informatics Program, Boston Children's Hospital, Boston, MA 02115, USA; Department of Biological Chemistry and Molecular Pharmacology, Harvard Medical School, Boston, MA 02115, USA; Computational Health Informatics Program, Boston Children's Hospital, Boston, MA 02115, USA; Stem Cell Program, Division of Hematology/Oncology, Boston Children's Hospital, Boston, MA 02115, USA; Department of Biological Chemistry and Molecular Pharmacology, Harvard Medical School, Boston, MA 02115, USA; Department of Pediatrics, Harvard Medical School, Boston, MA 02115, USA; Harvard Initiative for RNA Medicine, Boston, MA 02115, USA; Harvard Stem Cell Institute, Cambridge, MA 02138, USA

## Abstract

Ribosome profiling (Ribo-seq) is a powerful technology for globally monitoring RNA translation; ranging from codon occupancy profiling, identification of actively translated open reading frames (ORFs), to the quantification of translational efficiency under various physiological or experimental conditions. However, analyzing and decoding translation information from Ribo-seq data is not trivial. Although there are many existing tools to analyze Ribo-seq data, most of these tools are designed for specific or limited functionalities and an easy-to-use integrated tool to analyze Ribo-seq data is lacking. Fortunately, the small size (26–34 nt) of ribosome protected fragments (RPFs) in Ribo-seq and the relatively small amount of sequencing data greatly facilitates the development of such a web platform, which is easy to manipulate for users with or without bioinformatic expertise. Thus, we developed RiboToolkit (http://rnabioinfor.tch.harvard.edu/RiboToolkit), a convenient, freely available, web-based service to centralize Ribo-seq data analyses, including data cleaning and quality evaluation, expression analysis based on RPFs, codon occupancy, translation efficiency analysis, differential translation analysis, functional annotation, translation metagene analysis, and identification of actively translated ORFs. Besides, easy-to-use web interfaces were developed to facilitate data analysis and intuitively visualize results. Thus, RiboToolkit will greatly facilitate the study of mRNA translation based on ribosome profiling.

## INTRODUCTION

Ribosome profiling (Ribo-seq), also known as ribosome footprinting, has revolutionized the ‘translatomics’ field by mapping the position of ribosome-protected fragments (RPFs), which typically range in length from 26 to 34 nucleotides (nt), over the entire transcriptome (1,2). The scientific community has employed Ribo-seq to answer a wide range of questions, ranging from the identification of translated open reading frames (ORFs) to the quantification of relative translational efficiencies, while gaining precious mechanistic insight into the mRNA translation process (3). Translation is the bridge between RNA and protein, which is highly interconnected and subject to extensive, multi-step, post-transcriptional regulation, including pre-mRNA splicing, small RNA-mediated regulation, mRNA turnover, mRNA modifications, as well as many other mechanisms of translational control (4,5). More and more investigators are beginning to use Ribo-seq in their research to study various processes of post-transcriptional gene regulation.

Although there are already many existing tools to analyze Ribo-seq data, such as riboSeqR (6), Plastid (7), RUST (8), mQC (9), RiboProfiling (10), riboWaltz (11), GWIPS-viz (12), RiboVIEW (13) and Trips-Viz (14) for checking quality and visualizing RPF distribution and codon level statistics; RibORF (15), RiboTaper (16), ORF-RATER (17), SPECtre (18), riboHMM (19), RpBp (20), PRICE (21), RiboWave (22) and RiboCode (23) for translated ORF identification; Riborex (24), scikit-ribo (25), Anota (26), Babel (27), RiboDiff (28) and Xtail (29) for differential translation analysis, they are all designed for specific or limited functionalities. An easy-to-use tool to analyze mRNA translation in an integrated way is still lacking. Since Ribo-seq can provide diverse kinds of useful information about mRNA translation and each kind of analysis needs specific skills, there is a high demand among the RNA research community for such a one-stop tool to help them analyze Ribo-seq data in an integrated manner, not only for bioinformatics experts but also for the less bioinformatically inclined researchers. Fortunately, the small size of RPFs (26–34 nt) and the relatively small amount of sequencing data produced, greatly facilitate the development of such a convenient web server, which can be very easy to manipulate for users.

Here we present, RiboToolkit (http://rnabioinfor.tch.harvard.edu/RiboToolkit and https://bioinformatics.sc.cn/RiboToolkit), the first integrated web server for Ribo-seq data analysis, that we developed with these main functionalities: (i) data quality control by filtering low quality sequence reads and distinguishing RPFs from tRNA, snRNA, and rRNA tags; (ii) RPFs length distribution, coding frame distribution, and 3-nt periodicity analyses for Ribo-seq quality evaluation; (iii) codon usage and ribosome stalling analyses were designed to identify highly active codons and codon stalling events; (iv) actively translated ORFs can be efficiently identified with higher speed; (v) unbiased mRNA translation efficiency and differential translation analysis; (vi) functional annotation of differentially translated mRNAs can be performed using various gene functional datasets; (vii) metagene analysis designed to show the RPFs distribution for entire translatome; (viii) reproducibility analyses between replicates can be performed based on RPF expression, gene expression, and codon occupancy; (ix) RPF mapping can be interactively visualized on the webpage based on IGV.js; (x) CodonFreq tool was developed to study the codon constitution among different gene groups; (xi) supports different ways of data uploading, including collapsed FASTA and data web links; (xii) very user-friendly web interfaces and a convenient data analysis queuing system was developed; (xiii) the results can be flexibly exported in different formats; (xiv) mRNA translation can be studied for as many as 16 model species (Supplementary Table S1). Therefore, RiboToolkit is a very comprehensive and convenient tool for Ribosome profiling and will greatly benefit the study of mRNA translation.

## RiboToolkit WORKFLOW

**Figure 1.**
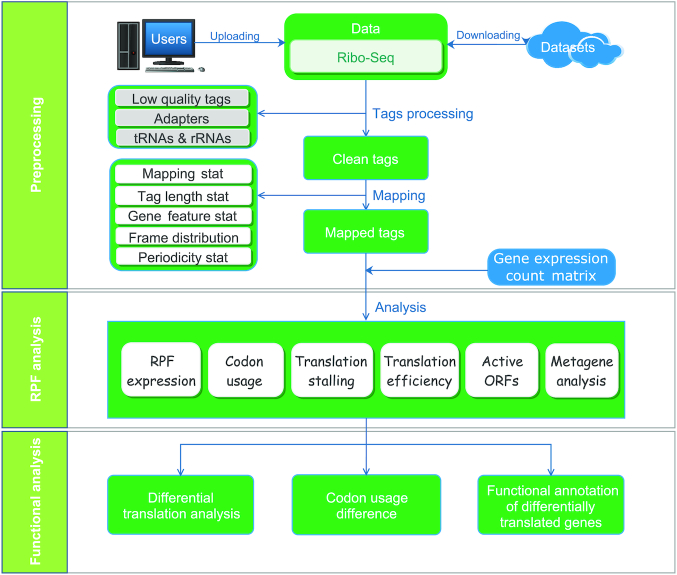
Overall RiboToolkit workflow.

RiboToolkit was constructed based on diverse data sources (Supplementary Table S1) and algorithms. tRNA sequences were downloaded from the GtRNAdb database (30). rRNA and snRNA sequences were retrieved from noncoding RNA annotations in Ensembl Genomes database (31). Protein coding gene sequences and gene annotations were downloaded from GENCODE database (32) for human (V19 and V32 for hg19 and hg38, respectively) and mouse (M23), and Ensembl Genomes database (31) for other species (Supplementary Table S1). The overall workflow contains three major parts: (i) Ribo-seq data pre-processing; (ii) RPF mapping and sequences analyses; and (iii) differential translation and functional analyses (Figure 1).

The uploaded sequences were first aligned to rRNAs, tRNA, and snRNA to exclude the RPFs coming from rRNA, tRNA, and snRNA using Bowtie v1.2.2 (33) with a maximum of two mismatches (-v 2) by default. Cleaned RPF sequences were then mapped to the reference genome using STAR v2.7.3a (34) with parameters (–outFilterMismatchNmax 2 –quantMode TranscriptomeSAM GeneCounts –outSAMattributes MD NH –outFilterMultimapNmax 1) by default. The unique genome-mapped RPFs are then mapped against protein coding transcripts using bowtie v1.2.2 with parameters ‘-a -v 2’ by default (33). Coding frame distribution and 3-nt periodicity analyses for Ribo-seq quality evaluation are performed based on riboWaltz v1.1.0 (11). The featureCounts program in the Subread package v1.6.3 (35) is used to count the number of RPFs uniquely mapped to CDS regions based on genome mapping file (-t CDS -g gene_id), which were then normalized as RPF Per Kilobase per Million mapped RPFs (RPKM). For codon-based analyses, 5′ mapped sites of RPFs (26–32 nt by default) translated in 0-frame were used to infer the P-sites with the offsets, which can be set by users or calculated based on the RPF mapping distribution around translation start sites using psite function in plastid v0.4.8 (7). The codon occupancy was further normalized by the basal occupancy which was calculated as the average occupancy of +1, +2, and +3 position downstream of A-sites (36). Pause score is further used to evaluate codon pause events using PausePred local version with default parameters (37). The upstream and downstream sequences (±50 nt) around pause sites were extracted from transcript sequences and different sequence features were calculated, including RNA secondary structure, minimum free energy (MFE), and GC content. RNA secondary structure and minimum free energy were calculated using RNAfold program in ViennaRNA Package v2.0 (38) with default parameters. For actively translated ORF identification, RPF reads mapped to the genome in end-to-end mode were extracted by removing the soft clipped reads from the BAM file generated by STAR, then RiboCode v1.2.11 (23), which shows high speed and sensitivity for annotating ORF (23), was used to identify all actively translated ORFs. In this process, RiboCode first constructs the candidate ORF library based on the constitution of start codons and stop codons on different transcripts (including both protein coding and non-coding RNAs). The actively translated ORFs were then identified by evaluating the statistically significant 3-nt periodicity (*P*-value < 0.05 by default) in each candidate ORF based on the distribution of RPFs in each frame.

**Figure 2.**
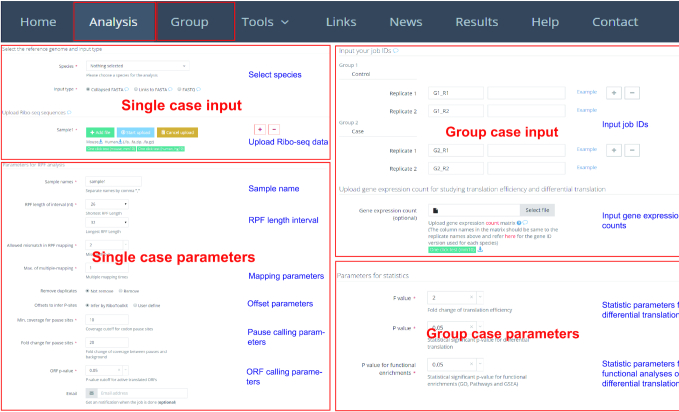
Inputs of RiboToolkit. In single case, RiboToolkit utilizes collapse FASTA file of Ribo-seq data as input. In group case, the job IDs of single case module are required as inputs and each group should contain at least one sample. The gene expression count matrix file of according input samples (RNA-Seq) is required to perform differential translation analysis.

The translation efficiency was calculated as the ratio between CDS RPF abundance and mRNA abundance for each gene, for which gene expression matrix (raw read counts) needs to be uploaded by the users in the group case web page (Figure 2). The gene expression count matrix is generated by merging raw read counts from accompanying RNA-seq data for different samples. The users can use many tools to count the reads from mapping BAM files of RNA-seq data, such as featureCounts (35) and HTseq (39). RiboToolkit provides the information and download links of gtf files used for each species. The difference in translation efficiency between two groups with more than two replicates is analyzed using Riborex v2.4.0 (24) based on DESeq2 engine, which models a natural dependence of translation on mRNA levels as a generalized linear model (40). For two groups without replicates, only fold change is calculated. To explore the biological implication of differentially translated genes (Fold change > 1.5 and adjust *P*-value < 0.05 by default), various functional gene enrichments are performed, including: (i) Gene Ontology (GO) and KEGG pathway from clusterProfiler package v3.14.3 (41) for all supported species; (ii) Reactome pathway from ReactomePA packages v1.30.0 (42) for human, mouse, rat, zebrafish, fly, and *Caenorhabditis elegans*; (iii) Disease Ontology, Network of Cancer Gene, DisGeNET disease genes from DOSE packages v3.12.0 (43) for human, mouse, rat, zebrafish, fly, and *C. elegans*. Meanwhile, Gene Set Enrichment Analysis (GSEA) for GO, KEGG and MSigDB functional gene sets (44) are supported for human, mouse, rat, zebrafish, fly and *C. elegans*. In the functional enrichment process, Fisher's exact test is used to perform enrichment analysis, while for the GSEA analysis, clusterProfiler package (41) is utilized. FDR < 0.05 was set as the statistically significant level by default.

## WEB SERVER INPUTS

The single case module allows users to submit Ribo-seq data or accessible web links to the data. The Ribo-seq data are required in collapsed FASTA format or as collapsed FASTA file further compressed in zip or gz format to accelerate the uploading. The header in the collapsed FASTA format likes ‘>seq1_x160’, where ‘seq1’ is a user-definable unique ID, while ‘160’ represents the frequency of RPFs of ‘seq1’. Meanwhile, batch submission of multiple samples is supported by clicking ‘add’ icon in the web page. The collapsed FASTA format can sharply reduce the size of FASTQ format files. For instance, a gzip compressed FASTQ file with 1.6 Gb size can be converted to a compressed collapsed FASTA file of 38 Mb size. Meanwhile, RiboToolkit (server #1) also provides users the options to upload FASTQ file, although the uploading speed is much slower than uploading collapsed FASTA. There is up to 5 Gb maximum upload size restriction. For the FASTQ file uploading, the adapter information is required, including 3′ adapter, 5′ adapter (optional), maximum allowed mismatches or match error rate, minimum overlap length between read and adapter, number of nucleotides clipped from both ends, and number of rounds for adapter trimming. After submission of data, the analysis queue system will provide the users with job IDs (a string with 16 characters) that can be used to retrieve the results once the job is finished.

In the group case module, the job IDs of single case module are required as inputs and each group should contain at least one sample. During the data analysis process, the web server will retrieve corresponding results for the jobs automatically. When the gene expression matrix file (raw read counts) of according input samples (RNA-Seq) is provided in group case, RiboToolkit will perform the translation efficiency calculation, differential translation analysis, and functional annotation of differentially translated genes. The gene expression count matrix can be generated by merging the raw count outputs from many tools, such as featureCount (35) and HTseq (39). The codonFreq tool can be used to perform codon enrichment analysis and compare the codon frequencies in the user's submitted genes compared with other background genes. Users can define a codon subset by inputting codon list in the web page.

RiboToolkit provides several flexible parameters for the users. A length interval can be set in advance and only the RPF sequences within this interval (26–32 nt by default) will be considered for downstream analyses. Meanwhile, RiboToolkit provides many other useful parameters (Figure 2), such as the number of allowed mismatches (with the default a maximum of two mismatches), maximum of multiple-mapping (unique mapping by default) in RPF sequence mapping, offsets to infer P-sites (calculated by psite function or inputted by users), minimum of RPF coverage, fold change compared with background for codon pause site identification, and *P*-value for actively translated ORF calling. For Ribo-seq data which use unique molecular identifier (UMI) for PCR duplication elimination (45), the algorithm implemented in RiboFlow-RiboR-RiboPy (46) and UMI-Reducer (https://github.com/smangul1/UMI-Reducer) are used to remove the PCR duplication. To detect differential translation between samples, the desired statistical significance of interest with *P*-value threshold and fold change in normalized sequence counts can be defined by users. The statistically significant level for functional enrichments of differentially translated genes can be set by users. In the codonFreq tool, the users can set the *P*-value threshold to define the codon enrichment between the codon frequency of the gene and genome background. All input webpages are organized with examples to help users achieve correct inputs.

## WEB SERVER OUTPUTS

**Figure 3.**
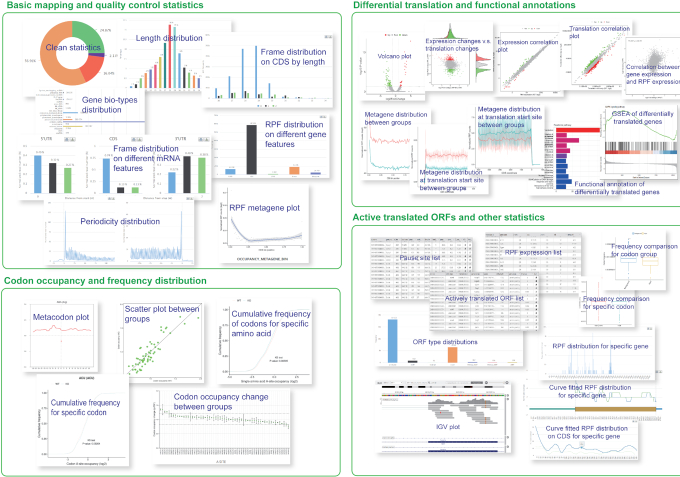
Screenshots of RiboToolkit outputs. The outputs typically contain four types of information: basic mapping and quality controls statistics, codon occupancy and frequency distribution, differential translation and functional annotations, and actively translated ORFs and other statistics.

All RiboToolkit outputs are presented in intuitive web interfaces, which typically contain the following information: (i) basic statistics of RPF tags, including RPF cleaning statistics by mapping to different potential contamination RNA types (rRNA, tRNA and snRNA) (46), RPF length distribution, RPF distribution on different gene biotypes (protein coding, lincRNA, antisense RNA, etc.) and RPF distribution on different gene features (5′ UTR, CDS, 3′ UTR, etc.); (ii) Ribo-seq quality statistics, including RPF coding frame distribution (frame 0, 1 and 2) on 5′ UTR, CDS and 3′ UTR, respectively, RPF coding frame distribution with different RPF length, RPF mapping around start codon for P-site inferring, RPF metagene distribution around translation start/end sites for 3-nt periodicity checking, and metagene coverage plots for whole CDS, CDS start region (300 bp) and CDS end region (300 bp); (iii) codon occupancy statistics, including codon occupancies of E, P, A, A +1, A + 2 and A + 3 sites; (iv) metaplot for individual codon; (v) Gene expression table from RPF counts, including RPF counts and RPKM values for 5′ UTR, CDS, 3′ UTR and whole mRNA; (vi) codon pause score and sequence context information (RNA secondary structure, minimum free energy, and GC content for both upstream and downstream sequences) for codon pausing sites; (vii) Actively translated ORF statistics, including actively translated ORF distribution plot and table of detailed ORF list. All the ORFs with statistically significant 3-nt periodicity distribution (*P*-value < 0.05 by default) are reported in the table. The users can further filter the ORF list by using RPF raw number or normalized RPF number to identify high confidence ORFs from the full list (Figure 3).

In group case study, the outputs contain: (i) a heatmap of RPF length distribution for all the samples in two groups; (ii) reproducibility analyses using RPF and gene expression, including correlation scatter plots between different replicates, PCA analysis and correlograms for different samples; (iii) codon occupancy scatter plot for A site between two groups and correlation plots among replicates in each group; (iv) codon occupancy changes for E, P, A, A +1, A + 2 and A + 3 sites between two groups; (v) cumulative codon occupancy plot for individual codons; (vi) cumulative codon occupancy plot for individual amino acids; (vii) the statistic plots of expression and translation changes, including the volcano plot of differential translation, the scatter plot of translation efficiency changes versus expression changes, gene expression scatter plot between two groups, translation efficiency scatter plot between two groups, the correlation plot between normalized RPF count and normalized gene counts; (viii) RPF metagene distribution between two groups; (ix) RPF metagene distribution for translation up-regulated and down-regulated, respectively; (x) differentially translated gene list; (xi) functional enrichment barplots and detailed lists of enriched terms for both translational up-regulated genes and down-regulated genes and (xii) GSEA result list. In codonFreq tool, the output includes the difference of total codon frequency between input genes and background genes, boxplots of codon frequency of each codon and the table of codon frequencies and enrichments for uploaded genes (Figure 3).

For each table in the results web pages, more detailed gene information (including sequence lengths for 5′UTR, CDS, 3′UTR, and whole transcript) are provided for each gene, which can be downloaded by clicking the ‘download CSV’ button located above the table. For each interactive plot generated using Highcharts JavaScript library (https://www.highcharts.com), RiboToolkit provides links (above the plot) to download the data for the plots in both txt and csv formats. Meanwhile, the users can download all the results in the tables and figures using ‘Download the results’ button in the front of result pages.

## COMPARISON WITH OTHER INTEGRATED TOOLS

There is a wide range of publicly available tools for Ribo-seq data analysis. However, to the best of our knowledge, the focus of many available tools is directed towards actively translated ORF identification, such as RibORF (15), RiboTaper (16) and ORF-RATER (17). Some tools are designed specifically for visualizing RPF distribution and codon statistics, such as riboWaltz (11), GWIPS-viz (12) and Trips-Viz (14). Other tools, such as Riborex (24) and Xtail (29), focus on differential translation efficiency analysis. Although integrated tools are designed for Ribo-seq analysis, including RiboTools (47), riboSeqR (6), Plastid (7), RiboProfiling (10), PROTEOFORMER (48,49), systemPipeR (50), RiboVIEW (13), riboStreamR (51), RiboFlow-RiboR-RiboPy (46) and RiboGalaxy (52), these tools provided just a limited number of functionalities and/or required many bioinformatics expertise to install, configure and manipulate them (Supplementary Table S2). Although RiboGalaxy provides many functions on the Galaxy web, but they are not integrated with each other. RiboToolkit is the first integrated one-stop web server for Ribo-seq data analysis (Supplementary Table S2): which provides many useful functionalities: (i) data quality control; (ii) Ribo-seq quality evaluation; (iii) Codon usage and ribosome stalling analyses, (iv) Actively translated ORFs identification; (v) gene translation efficiency and differential translation analysis; (vi) differential translation gene functional annotation based on various functional sets; (vii) RPF metagene analysis for CDS region and translation start/end sites; (viii) interactive visualization of RPF mapping on the web page; (ix) CodonFreq tool was developed to study the codon constitution of different gene groups; (x) different ways of data uploading; (xi) very user-friendly web interfaces and a convenient data analysis queuing system; (xii) RNA translation can be studied for as many as 16 species.

## CASE STUDIES

**Figure 4.**
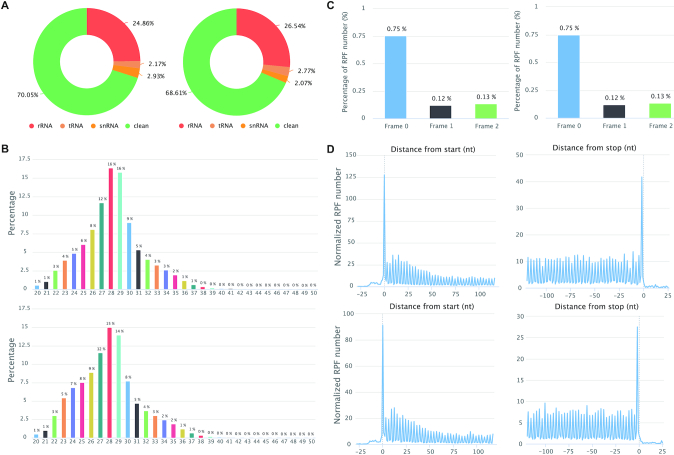
RiboToolkit quality control outputs for Ribo-seq data of Mettl1 knockout and control mESCs. (**A**) RPF mapping statistics (rRNA, tRNA, snRNA and clean sequences). (**B**) RPF length distribution. (**C**) RPF frame distribution. Y-axis indicates the percentage of RPFs in each coding frame while x-axis represents different coding frames (frame 0, frame 1, and frame 2). (**D**) global 3-nt periodicity checking using metagene distribution plot.

**Figure 5.**
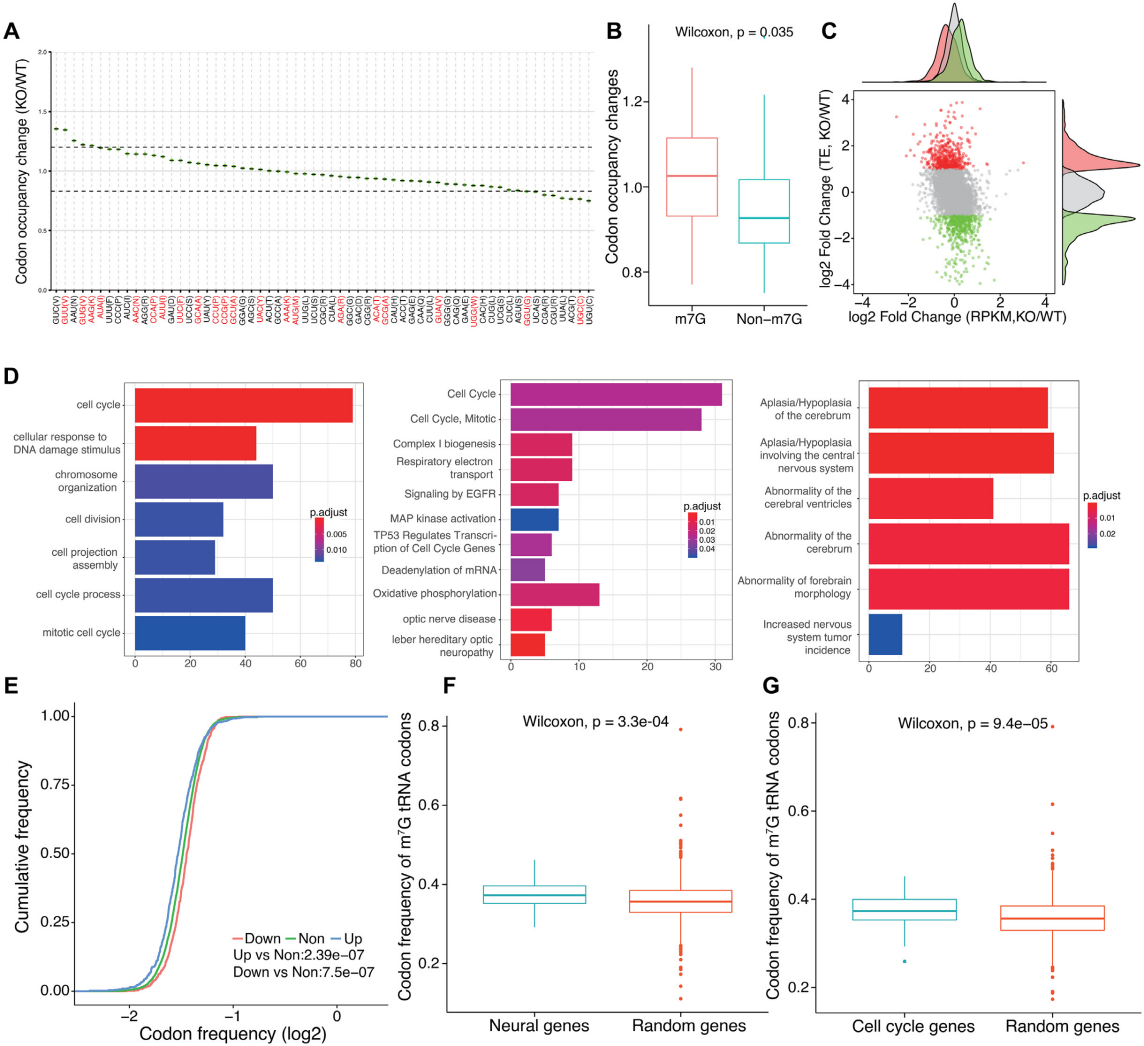
RiboToolkit analysis reveals significant changes in codon occupancy and mRNA translation in Mettl1 knockout. (**A**) Codon occupancy changes between Mettl1 knockout and wild type. (**B**) The boxplot of codon occupancy changes comparing codons decoded by m^7^G tRNAs and other codons. (**C**) The scatter plot of translation efficiency changes versus gene expression changes. The red and green dots represent translationally up-regulated and down-regulated genes (≥2-fold changes), respectively. Many genes showed differential translation in Mettl1 knockout mESCs while the global mRNA expression changes are limited compared with the changes in mRNA translation. (**D**) Functional enrichments of differentially translated genes. (**E**) Cumulative distribution of codon frequencies among up-regulated, down-regulated, and non-differentially translated mRNAs. (**F**) Codon frequency of m^7^G tRNA decoded codons in neural genes. (**G**) Codon frequency of m^7^G tRNA decoded codons in cell cycle genes.

Transfer RNAs (tRNAs) are subjected to numerous RNA modifications, which can directly control their folding and stability. *N*^7^-Methylguanosine (m^7^G) at nucleotide 46 (m^7^G46) is one of the most prevalent modifications and has important physiological functions in mammals. A total of 22 m^7^G modifications were identified in mouse embryonic stem cells (mESCs) and knockout of METTL1 was shown to greatly decrease the stability of 22 m^7^G tRNAs and further impact mRNA translation of cell cycle and neurodevelopmental genes (53). RiboToolkit was used to study the translation changes based on Ribo-seq data of Mettl1 knockout and control in mouse embryonic stem cells (mESCs) (GSE112670, Supplementary Table S3) (53). The mapping statistics, RPF periodicity, RPF length distribution, and metagene plot by RiboToolkit confirmed the good quality of the Ribo-seq data (Figure 4). Codon occupancy analysis confirmed that the majority of m^7^G-modified tRNAs decoded codons showed significantly higher occupancy than codons that are decoded by tRNAs that are not m^7^G-modified (Figure 5A and B). Translation efficiency analysis by RiboToolkit showed that the translation is obviously impacted upon knocking out Mettl1 compared with the mRNA expression level changes (Figure 5C). Codon frequency distribution from RiboToolkit indicated that the frequency of m^7^G tRNAs decoded codons are significantly enriched in translation down-regulated genes (Figure 5E). The functional annotation of Gene Ontology and various pathways by RiboToolkit showed that cell cycle and neural genes are significantly enriched among the translationally down-regulated genes (Figure 5D), which are consistent with the original findings. Further analyses also confirmed the significant higher m^7^G codon frequencies of cell cycle genes and neural genes compared with random background genes (Figure 5F and G).

Certain yeast strains show a large proportion of sites with high codon occupancy due to a high abundance of paused ribosomes (54). Yeast treated with 3-amino-1,2,4-triazole (3-AT), an inhibitor of histidine biosynthesis, can induce ribosome pausing. Based on the Ribo-seq data of 3-AT treatment in Yeast (GSE52968, Supplementary Table S3) (54). RiboToolkit analyses showed a significant shift in a cumulative distribution of pause scores and a peak in metagene distribution plot, indicating the significant pauses at histidine codons (Supplementary Figure S1). In yeast, the wobble uridine (U34) in tRNA wobble nucleoside is almost always modified and can enhance codon recognition and binding (36). RiboToolkit analyses based on ribosome profiling data of ncs2Δelp6Δ yeast mutant (lacking all U34 modifications) and wide type (GSE67387, Supplementary Table S3) (36) revealed strikingly distinct effects of U34 modification loss on ribosome occupancy. CAA and AAA codons, decoded by the mcm^5^s^2^U34-containing tRNA-UUG and tRNA-UUU, were enriched within the A-site in the mutant (Supplementary Figure S2A), suggesting they are translated more slowly when U34 modification is depleted or attenuated. For other codons, including GAA, which is also decoded by a mcm^5^s^2^U34-containing tRNA, the effect on ribosome occupancy is modest. The comparison of the A-site ribosome occupancy at individual codons in wild type and ncs2Δelp6Δ mutant showed that a significantly larger proportion of CAA and AAA codons had high occupancy in mutant (Supplementary Figure S2B), indicative of widespread translational slowdown. By contrast, single-codon A-site occupancy change at GAA was not significant in the two strains, consistent with the global codon occupancy measurements (Supplementary Figure S2B).

**Figure 6.**
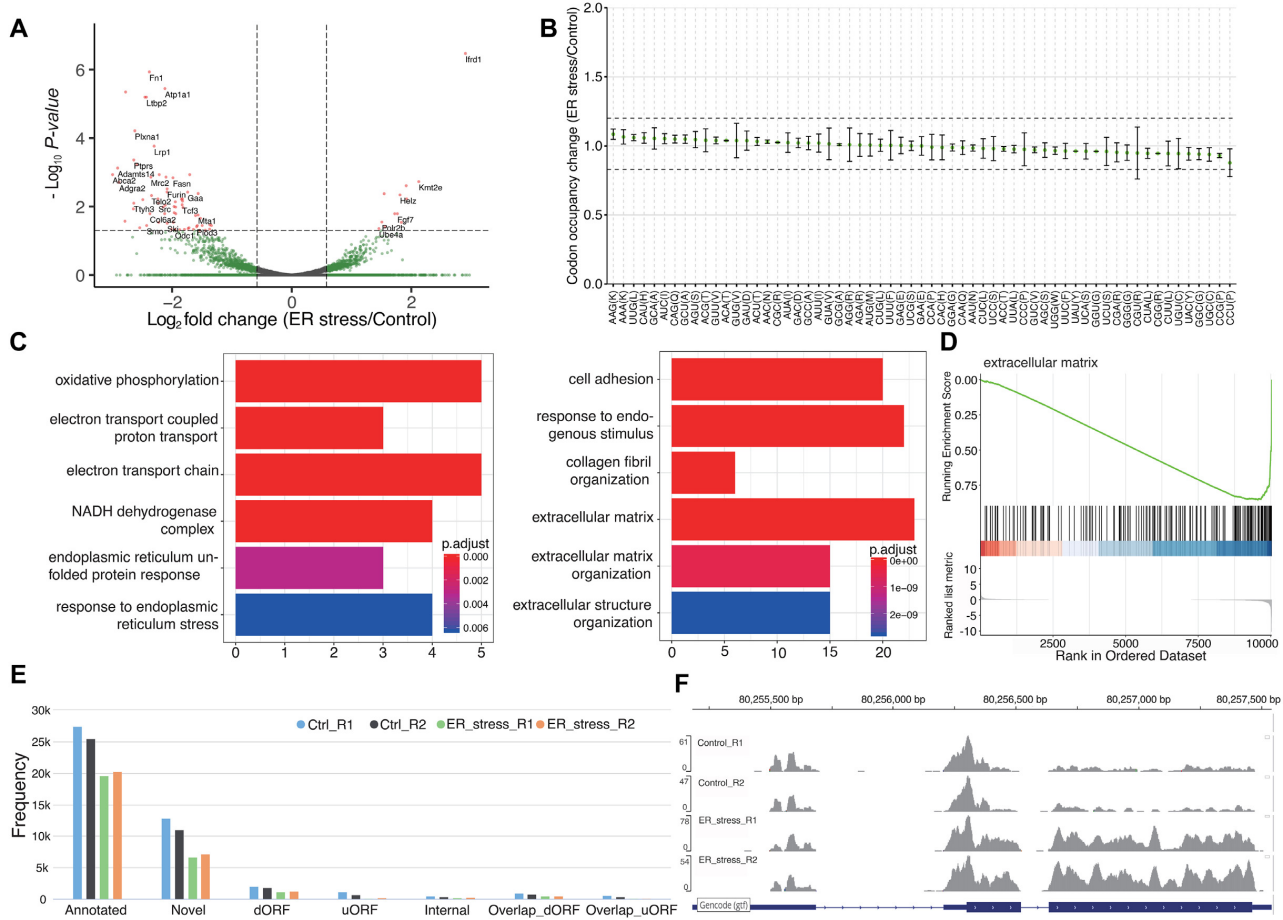
RiboToolkit analysis shows that ER stress induces differential translation of many mRNAs but limited changes in codon occupancy. (**A**) Volcano plot of differentially translated genes between ER stress conditions (Thapsigargin-treated) and controls (DMSO-treated). (**B**) Codon occupancy changes is limited between ER stress conditions and controls. (**C**) GO and pathways functional enrichments of differentially translated genes. (**D**) GSEA of differentially translated genes further indicates that extracellular matrix organization is affected under ER stress. (**E**) Statistics of actively translated ORF among differential samples. (**F**) Alt4, which is translational up-regulated, is used as example to show the RPF mapping in IGV.js.

Endoplasmic reticulum (ER) stress impacts translation (55). We used RiboToolkit to systematical profile translation in ER-stress conditions of NIH3T3 cells (GSE103667, Supplementary Table S3) (56). Translation efficiency indicates that a total of 120 genes are significantly differentially translated (fold change > 1.5 and adjust *P*-value < 0.05). There are many more down-regulated genes compared with up-regulated genes (91 versus 29) (Figure 6A). The up-regulated gene includes Atf4, a transcriptional factor, which is well known from other studies to be translationally up-regulated upon ER stress (57,58) (Figure 6F). Codon occupancy analysis indicated that the global translation on specific codons is not affected under the ER-stress condition (Figure 6B). Functional annotation of differentially translated genes revealed significant enrichment in oxidative phosphorylation, electron transport chain, endoplasmic reticulum unfolded protein response, response to endoplasmic reticulum stress, cell adhesion, and extracellular matrix, etc. GSEA results also indicate the significant association between ER-stress and extracellular matrix function (Figure 6C and D). Active ORF analysis showed in NIH3T3 cells that most ORFs come from known CDS region (annotated ORF). There are however many other ORFs identified, including uORF (upstream ORF), overlapping uORF, dORF (downstream ORF), overlapping dORF (translation read through), internal ORF (ORF on CDS with different coding frame or frame shift) and novel ORF (Figure 6E).

**Figure 7.**
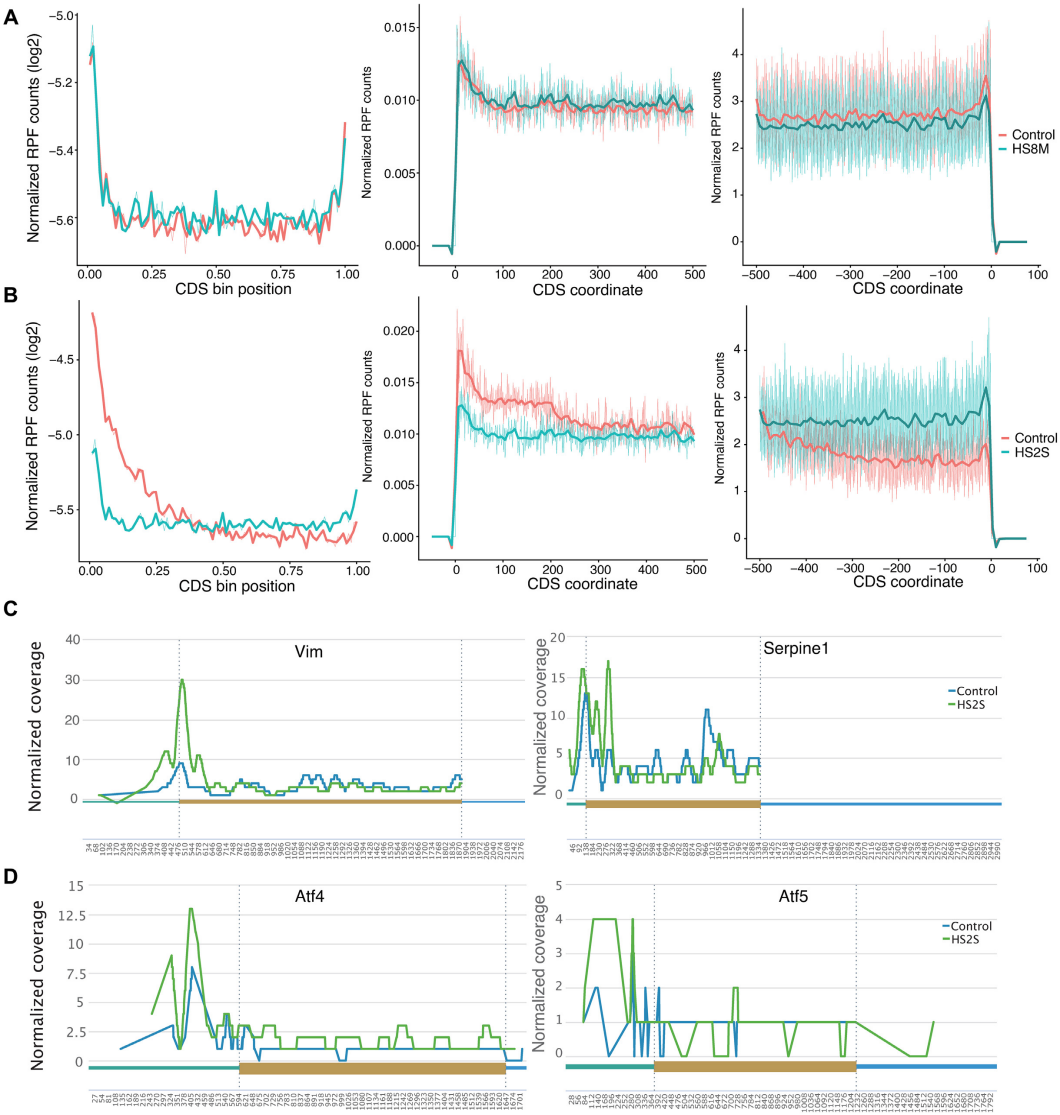
RPF metagene distribution from RiboToolkit analysis indicates that mRNA translation is globally impacted in mouse 3T3 fibroblast cells in response to acute heat shock. (**A**) RFP metagene distribution plots (whole CDS and regions around translation start/end sites) comparing cells that experienced chronic heat stress (42°C for 8 h, HS8M) and control cells. (**B**) RFP metagene distribution plots comparing cells that experienced acute heat stress (44°C for 2 h, HS2S) and control cells. (**C**) Numerous individual mRNAs with sufficient RPF coverage showed a similar distribution to the RPF metagene plot, such as Vim and Serpine1. (**D**) There are exceptions where some genes escape from the global elongation and initiation blocks, such as Atf4 and Atf5, which were translationally up-regulated via translation of uORFs.

Global repression of protein synthesis occurs during heat shock response and has been attributed primarily to inhibition of translation initiation (59). RiboToolkit was used to study global translational regulation during chronic heat stress (42°C for 8 h, HS8M) and acute heat stress (44°C for 2 h, HS2S) in mouse 3T3 fibroblast cell (GSE32060, Supplementary Table S3) (59). Metagene RPF distribution analysis showed that change is generally modest in response to chronic heat stress (Figure 7A), while a dramatic change in relative ribosome occupancy occurs in response to acute heat stress, especially at the translation initiation region (∼200 nt after the initiation site) (Figure 7B), which may indicate translation regulation after initiation. Numerous individual genes with sufficient RPF coverage showed a similar distribution to the RPF metagene plot, such as Vim and Serpine1 (Figure 7C). There are exceptions with some genes escaping from the global elongation and initiation blocks, such as Atf4 and Atf5 (Figure 7D), two important transcription factor genes that regulate responses to a variety of stress conditions (55,58). Both of these factors have been revealed to be translationally up-regulated under stress conditions via a mechanism involving translation of uORFs (Figure 7D) (55,57,60).

## DATA UPLOAD SPEED AND ANALYSIS SPEED EVALUATION

To test the data upload and analysis efficiency, we uploaded the dataset used as case studies (Supplementary Table S3) to the two high-performance computer servers from both the USA and China. The compressed file sizes of samples GSE103667 (four samples), GSE67387 (two samples), GSE52968 (two samples), GSE112670 (two samples), and GSE32060 (3 samples) were 97, 289, 67, 44 and 78MB, respectively. The upload speed of server #1 (rnabioinfor.tch.harvard.edu) is faster than server #2 (bioinformatics.sc.cn), while the data analysis speed is slower than server #2 (Supplementary Table S3). The upload speed also depends upon the web condition when uploading the data. It is expected that the analysis speed is higher in server #2 due to its better hardware.

## IMPLEMENTATION

The web servers are hosted within a Linux system containing PHP/Apache environment. server #1 is equipped with 16 cores Intel Xeon E7440 (2.4 GHz) and 128GB of RAM while server #2 is equipped with four hexadeca-core (64 cores) Intel Xeon processors (2.1 GHz each) and 512GB of RAM. The back-end pipeline is implemented in the Perl/R language. The bitmap plots in PNG and PDF formats are drawn by R (http://www.r-project.org) packages, including ggplot2, cowplot, and ggpubr. The visualization web interfaces are created using several JavaScript libraries, including JQuery, Bootstrap.js, DataTable.js, Highchart.js and igv.js libraries, etc. which provide users with a highly dynamic, interactive, and intuitive interfaces for manipulating the software and viewing the analysis results.

## CONCLUSIONS

Ribosome profiling (Ribo-seq) is proven as a very powerful technology for globally monitoring mRNA translation and more and more laboratories have started using this powerful approach in their studies. However, a convenient and integrated tool for Ribo-seq data is still lacking. In this study, RiboToolkit, the first integrated one-stop web-based toolkit, was developed to analyze ribosome profiling data for users with or without bioinformatics expertise. Various case studies validated the useful functionalities and reproducibility of RiboToolkit. Currently, it supports 16 model species, and additional reference genomes will be integrated in future updates. Moreover, additional functionality (such as ribosome drop-off detection (61,62)) and more input formats (such as BAM file) will be supported in RiboToolkit in the future. Due to the large size of RNA-seq data, RiboToolkit web server only supports the uploading of gene expression counts. The virtureBox or Docker versions of RiboToolkit will be developed in the future, which will support the inputs of accompanying RNA-seq data with the Ribo-seq data. Taken together, we believe that RiboToolkit will greatly facilitate translation studies based on ribosome profiling.

## Supplementary Material

gkaa395_Supplemental_File

## References

[1] Ingolia N.T., Ghaemmaghami S., Newman J.R., Weissman J.S. Genome-wide analysis in vivo of translation with nucleotide resolution using ribosome profiling. Science (New York, N.Y.). 2009; 324:218–223. 10.1126/science.1168978 PMC2746483 19213877

[2] Calviello L., Ohler U. Beyond Read-Counts: Ribo-seq data analysis to understand the functions of the transcriptome. Trends Genet. 2017; 33:728–744. 28887026 10.1016/j.tig.2017.08.003

[3] Brar G.A., Weissman J.S. Ribosome profiling reveals the what, when, where and how of protein synthesis. Nat. Rev. Mol. Cell Biol. 2015; 16:651–664. 26465719 10.1038/nrm4069 PMC5522010

[4] Choe J., Lin S., Zhang W., Liu Q., Wang L., Ramirez-Moya J., Du P., Kim W., Tang S., Sliz P. et al. mRNA circularization by METTL3-eIF3h enhances translation and promotes oncogenesis. Nature. 2018; 561:556–560. 30232453 10.1038/s41586-018-0538-8 PMC6234840

[5] Fabian M.R., Sonenberg N., Filipowicz W. Regulation of mRNA translation and stability by microRNAs. Annu. Rev. Biochem. 2010; 79:351–379. 20533884 10.1146/annurev-biochem-060308-103103

[6] Chung B.Y., Hardcastle T.J., Jones J.D., Irigoyen N., Firth A.E., Baulcombe D.C., Brierley I. The use of duplex-specific nuclease in ribosome profiling and a user-friendly software package for Ribo-seq data analysis. RNA. 2015; 21:1731–1745. 26286745 10.1261/rna.052548.115 PMC4574750

[7] Dunn J.G., Weissman J.S. Plastid: nucleotide-resolution analysis of next-generation sequencing and genomics data. BMC Genomics. 2016; 17:958. 27875984 10.1186/s12864-016-3278-x PMC5120557

[8] O’Connor P.B., Andreev D.E., Baranov P.V. Comparative survey of the relative impact of mRNA features on local ribosome profiling read density. Nat. Commun. 2016; 7:12915. 27698342 10.1038/ncomms12915 PMC5059445

[9] Verbruggen S., Menschaert G. mQC: A post-mapping data exploration tool for ribosome profiling. Comput. Methods Programs Biomed. 2019; 181:104806. 30401579 10.1016/j.cmpb.2018.10.018

[10] Popa A., Lebrigand K., Paquet A., Nottet N., Robbe-Sermesant K., Waldmann R., Barbry P. RiboProfiling: a Bioconductor package for standard Ribo-seq pipeline processing [version 1; peer review: 3 approved]. F1000Research. 2016; 5:1309. 27347386 10.12688/f1000research.8964.1 PMC4918025

[11] Lauria F., Tebaldi T., Bernabo P., Groen E.J.N., Gillingwater T.H., Viero G. riboWaltz: Optimization of ribosome P-site positioning in ribosome profiling data. PLoS Comput. Biol. 2018; 14:e1006169. 30102689 10.1371/journal.pcbi.1006169 PMC6112680

[12] Michel A.M., Fox G., A M.K., De Bo C., O’Connor P.B., Heaphy S.M., Mullan J.P., Donohue C.A., Higgins D.G., Baranov P.V. GWIPS-viz: development of a ribo-seq genome browser. Nucleic Acids Res. 2014; 42:D859–D864. 24185699 10.1093/nar/gkt1035 PMC3965066

[13] Legrand C., Tuorto F. RiboVIEW: a computational framework for visualization, quality control and statistical analysis of ribosome profiling data. Nucleic Acids Res. 2020; 48:e7. 31777932 10.1093/nar/gkz1074 PMC6954398

[14] Kiniry S.J., O’Connor P.B.F., Michel A.M., Baranov P.V. Trips-Viz: a transcriptome browser for exploring Ribo-Seq data. Nucleic Acids Res. 2019; 47:D847–D852. 30239879 10.1093/nar/gky842 PMC6324076

[15] Ji Z., Song R., Regev A., Struhl K. Many lncRNAs, 5′UTRs, and pseudogenes are translated and some are likely to express functional proteins. eLife. 2015; 4:e08890. 26687005 10.7554/eLife.08890 PMC4739776

[16] Calviello L., Mukherjee N., Wyler E., Zauber H., Hirsekorn A., Selbach M., Landthaler M., Obermayer B., Ohler U. Detecting actively translated open reading frames in ribosome profiling data. Nat. Methods. 2016; 13:165–170. 26657557 10.1038/nmeth.3688

[17] Fields A.P., Rodriguez E.H., Jovanovic M., Stern-Ginossar N., Haas B.J., Mertins P., Raychowdhury R., Hacohen N., Carr S.A., Ingolia N.T. et al. A Regression-Based analysis of Ribosome-Profiling data reveals a conserved complexity to mammalian translation. Mol. Cell. 2015; 60:816–827. 26638175 10.1016/j.molcel.2015.11.013 PMC4720255

[18] Chun S.Y., Rodriguez C.M., Todd P.K., Mills R.E. SPECtre: a spectral coherence–based classifier of actively translated transcripts from ribosome profiling sequence data. BMC Bioinformatics. 2016; 17:482. 27884106 10.1186/s12859-016-1355-4 PMC5123373

[19] Raj A., Wang S.H., Shim H., Harpak A., Li Y.I., Engelmann B., Stephens M., Gilad Y., Pritchard J.K. Thousands of novel translated open reading frames in humans inferred by ribosome footprint profiling. eLife. 2016; 5:e13328. 27232982 10.7554/eLife.13328 PMC4940163

[20] Malone B., Atanassov I., Aeschimann F., Li X., Grosshans H., Dieterich C. Bayesian prediction of RNA translation from ribosome profiling. Nucleic Acids Res. 2017; 45:2960–2972. 28126919 10.1093/nar/gkw1350 PMC5389577

[21] Erhard F., Halenius A., Zimmermann C., L’Hernault A., Kowalewski D.J., Weekes M.P., Stevanovic S., Zimmer R., Dolken L. Improved Ribo-seq enables identification of cryptic translation events. Nat. Methods. 2018; 15:363–366. 29529017 10.1038/nmeth.4631 PMC6152898

[22] Xu Z., Hu L., Shi B., Geng S., Xu L., Wang D., Lu Z.J. Ribosome elongating footprints denoised by wavelet transform comprehensively characterize dynamic cellular translation events. Nucleic Acids Res. 2018; 46:e109. 29945224 10.1093/nar/gky533 PMC6182183

[23] Xiao Z., Huang R., Xing X., Chen Y., Deng H., Yang X. De novo annotation and characterization of the translatome with ribosome profiling data. Nucleic Acids Res. 2018; 46:e61. 29538776 10.1093/nar/gky179 PMC6007384

[24] Li W., Wang W., Uren P.J., Penalva L.O.F., Smith A.D. Riborex: fast and flexible identification of differential translation from Ribo-seq data. Bioinformatics. 2017; 33:1735–1737. 28158331 10.1093/bioinformatics/btx047 PMC5860393

[25] Fang H., Huang Y.F., Radhakrishnan A., Siepel A., Lyon G.J., Schatz M.C. Scikit-ribo enables accurate estimation and robust modeling of translation dynamics at codon resolution. Cell Syst. 2018; 6:180–191. 29361467 10.1016/j.cels.2017.12.007 PMC5832574

[26] Larsson O., Sonenberg N., Nadon R. anota: analysis of differential translation in genome-wide studies. Bioinformatics. 2011; 27:1440–1441. 21422072 10.1093/bioinformatics/btr146

[27] Olshen A.B., Hsieh A.C., Stumpf C.R., Olshen R.A., Ruggero D., Taylor B.S. Assessing gene-level translational control from ribosome profiling. Bioinformatics. 2013; 29:2995–3002. 24048356 10.1093/bioinformatics/btt533 PMC3834798

[28] Zhong Y., Karaletsos T., Drewe P., Sreedharan V.T., Kuo D., Singh K., Wendel H.G., Ratsch G. RiboDiff: detecting changes of mRNA translation efficiency from ribosome footprints. Bioinformatics. 2017; 33:139–141. 27634950 10.1093/bioinformatics/btw585 PMC5198522

[29] Xiao Z., Zou Q., Liu Y., Yang X. Genome-wide assessment of differential translations with ribosome profiling data. Nat. Commun. 2016; 7:11194. 27041671 10.1038/ncomms11194 PMC4822032

[30] Chan P.P., Lowe T.M. GtRNAdb 2.0: an expanded database of transfer RNA genes identified in complete and draft genomes. Nucleic Acids Res. 2016; 44:D184–D189. 26673694 10.1093/nar/gkv1309 PMC4702915

[31] Hubbard T., Barker D., Birney E., Cameron G., Chen Y., Clark L., Cox T., Cuff J., Curwen V., Down T. et al. The Ensembl genome database project. Nucleic Acids Res. 2002; 30:38–41. 11752248 10.1093/nar/30.1.38 PMC99161

[32] Frankish A., Diekhans M., Ferreira A.M., Johnson R., Jungreis I., Loveland J., Mudge J.M., Sisu C., Wright J., Armstrong J. et al. GENCODE reference annotation for the human and mouse genomes. Nucleic Acids Res. 2019; 47:D766–D773. 30357393 10.1093/nar/gky955 PMC6323946

[33] Langmead B., Trapnell C., Pop M., Salzberg S.L. Ultrafast and memory-efficient alignment of short DNA sequences to the human genome. Genome Biol. 2009; 10:R25. 19261174 10.1186/gb-2009-10-3-r25 PMC2690996

[34] Dobin A., Davis C.A., Schlesinger F., Drenkow J., Zaleski C., Jha S., Batut P., Chaisson M., Gingeras T.R. STAR: ultrafast universal RNA-seq aligner. Bioinformatics. 2013; 29:15–21. 23104886 10.1093/bioinformatics/bts635 PMC3530905

[35] Liao Y., Smyth G.K., Shi W. featureCounts: an efficient general purpose program for assigning sequence reads to genomic features. Bioinformatics. 2014; 30:923–930. 24227677 10.1093/bioinformatics/btt656

[36] Nedialkova D.D., Leidel S.A. Optimization of codon translation rates via tRNA modifications maintains proteome integrity. Cell. 2015; 161:1606–1618. 26052047 10.1016/j.cell.2015.05.022 PMC4503807

[37] Kumari R., Michel A.M., Baranov P.V. PausePred and Rfeet: webtools for inferring ribosome pauses and visualizing footprint density from ribosome profiling data. RNA. 2018; 24:1297–1304. 30049792 10.1261/rna.065235.117 PMC6140459

[38] Lorenz R., Bernhart S.H., Honer Zu Siederdissen C., Tafer H., Flamm C., Stadler P.F., Hofacker I.L. ViennaRNA Package 2.0. Algorith. Mol. Biol.: AMB. 2011; 6:26. 10.1186/1748-7188-6-26 PMC3319429 22115189

[39] Anders S., Pyl P.T., Huber W. HTSeq–a Python framework to work with high-throughput sequencing data. Bioinformatics. 2015; 31:166–169. 25260700 10.1093/bioinformatics/btu638 PMC4287950

[40] Love M.I., Huber W., Anders S. Moderated estimation of fold change and dispersion for RNA-seq data with DESeq2. Genome Biol. 2014; 15:550. 25516281 10.1186/s13059-014-0550-8 PMC4302049

[41] Yu G., Wang L.G., Han Y., He Q.Y. clusterProfiler: an R package for comparing biological themes among gene clusters. Omics. 2012; 16:284–287. 22455463 10.1089/omi.2011.0118 PMC3339379

[42] Yu G., He Q.Y. ReactomePA: an R/Bioconductor package for reactome pathway analysis and visualization. Mol. Biosyst. 2016; 12:477–479. 26661513 10.1039/c5mb00663e

[43] Yu G., Wang L.G., Yan G.R., He Q.Y. DOSE: an R/Bioconductor package for disease ontology semantic and enrichment analysis. Bioinformatics. 2015; 31:608–609. 25677125 10.1093/bioinformatics/btu684

[44] Liberzon A., Birger C., Thorvaldsdottir H., Ghandi M., Mesirov J.P., Tamayo P. The Molecular Signatures Database (MSigDB) hallmark gene set collection. Cell Syst. 2015; 1:417–425. 26771021 10.1016/j.cels.2015.12.004 PMC4707969

[45] McGlincy N.J., Ingolia N.T. Transcriptome-wide measurement of translation by ribosome profiling. Methods. 2017; 126:112–129. 28579404 10.1016/j.ymeth.2017.05.028 PMC5582988

[46] Ozadam H., Geng M., Cenik C. RiboFlow, RiboR and RiboPy: an ecosystem for analyzing ribosome profiling data at read length resolution. Bioinformatics. 2020; 36:2929–2931. 31930375 10.1093/bioinformatics/btaa028 PMC7203755

[47] Legendre R., Baudin-Baillieu A., Hatin I., Namy O. RiboTools: a Galaxy toolbox for qualitative ribosome profiling analysis. Bioinformatics. 2015; 31:2586–2588. 25812744 10.1093/bioinformatics/btv174

[48] Crappe J., Ndah E., Koch A., Steyaert S., Gawron D., De Keulenaer S., De Meester E., De Meyer T., Van Criekinge W., Van Damme P. et al. PROTEOFORMER: deep proteome coverage through ribosome profiling and MS integration. Nucleic Acids Res. 2015; 43:e29. 25510491 10.1093/nar/gku1283 PMC4357689

[49] Verbruggen S., Ndah E., Van Criekinge W., Gessulat S., Kuster B., Wilhelm M., Van Damme P., Menschaert G. PROTEOFORMER 2.0: Further developments in the ribosome profiling-assisted proteogenomic hunt for new proteoforms. Mol. Cell. Proteomics: MCP. 2019; 18:S126–S140. 31040227 10.1074/mcp.RA118.001218 PMC6692777

[50] Backman T.W.H, Girke T. systemPipeR: NGS workflow and report generation environment. BMC Bioinformatics. 2016; 17:388. 27650223 10.1186/s12859-016-1241-0 PMC5029110

[51] Perkins P., Mazzoni-Putman S., Stepanova A., Alonso J., Heber S. RiboStreamR: a web application for quality control, analysis, and visualization of Ribo-seq data. BMC Genomics. 2019; 20:422. 31167636 10.1186/s12864-019-5700-7 PMC6551240

[52] Michel A.M., Mullan J.P., Velayudhan V., O’Connor P.B., Donohue C.A., Baranov P.V. RiboGalaxy: A browser based platform for the alignment, analysis and visualization of ribosome profiling data. RNA Biology. 2016; 13:316–319. 26821742 10.1080/15476286.2016.1141862 PMC4829337

[53] Lin S., Liu Q., Lelyveld V.S., Choe J., Szostak J.W., Gregory R.I. Mettl1/Wdr4-Mediated m(7)G tRNA methylome is required for normal mRNA translation and embryonic stem cell Self-Renewal and differentiation. Mol. Cell. 2018; 71:244–255. 29983320 10.1016/j.molcel.2018.06.001 PMC6086580

[54] Guydosh N.R., Green R. Dom34 rescues ribosomes in 3′ untranslated regions. Cell. 2014; 156:950–962. 24581494 10.1016/j.cell.2014.02.006 PMC4022138

[55] Zhou D., Palam L.R., Jiang L., Narasimhan J., Staschke K.A., Wek R.C. Phosphorylation of eIF2 directs ATF5 translational control in response to diverse stress conditions. J. Biol. Chem. 2008; 283:7064–7073. 18195013 10.1074/jbc.M708530200

[56] Namkoong S., Ho A., Woo Y.M., Kwak H., Lee J.H. Systematic characterization of Stress-Induced RNA granulation. Mol. Cell. 2018; 70:175–187. 29576526 10.1016/j.molcel.2018.02.025 PMC6359928

[57] Vattem K.M., Wek R.C. Reinitiation involving upstream ORFs regulates ATF4 mRNA translation in mammalian cells. PNAS. 2004; 101:11269–11274. 15277680 10.1073/pnas.0400541101 PMC509193

[58] Wortel I.M.N., van der Meer L.T., Kilberg M.S., van Leeuwen F.N. Surviving Stress: Modulation of ATF4-Mediated stress responses in normal and malignant cells. Trends Endocrinol. Metab. 2017; 28:794–806. 28797581 10.1016/j.tem.2017.07.003 PMC5951684

[59] Shalgi R., Hurt J.A., Krykbaeva I., Taipale M., Lindquist S., Burge C.B. Widespread regulation of translation by elongation pausing in heat shock. Mol. Cell. 2013; 49:439–452. 23290915 10.1016/j.molcel.2012.11.028 PMC3570722

[60] Starck S.R., Tsai J.C., Chen K., Shodiya M., Wang L., Yahiro K., Martins-Green M., Shastri N., Walter P. Translation from the 5′ untranslated region shapes the integrated stress response. Science (New York, N.Y.). 2016; 351:aad3867. 10.1126/science.aad3867 PMC4882168 26823435

[61] Sin C., Chiarugi D., Valleriani A. Quantitative assessment of ribosome drop-off in E. coli. Nucleic Acids Res. 2016; 44:2528–2537. 26935582 10.1093/nar/gkw137 PMC4824120

[62] Subramaniam A.R., Zid B.M., O'Shea E.K. An integrated approach reveals regulatory controls on bacterial translation elongation. Cell. 2014; 159:1200–1211. 25416955 10.1016/j.cell.2014.10.043 PMC4243059

