# Supplementary material for: RiboToolkit: an integrated platform for analysis and annotation of ribosome profiling data to decode mRNA translation at codon resolution
Source: Nucleic Acids Res. 2020 May 19;48(W1):W218–29. doi: 10.1093/nar/gkaa395 (PMC7319539; doi:10.1093/nar/gkaa395)
Supplement: gkaa395_Supplemental_File [file gkaa395_supplemental_file.pdf]

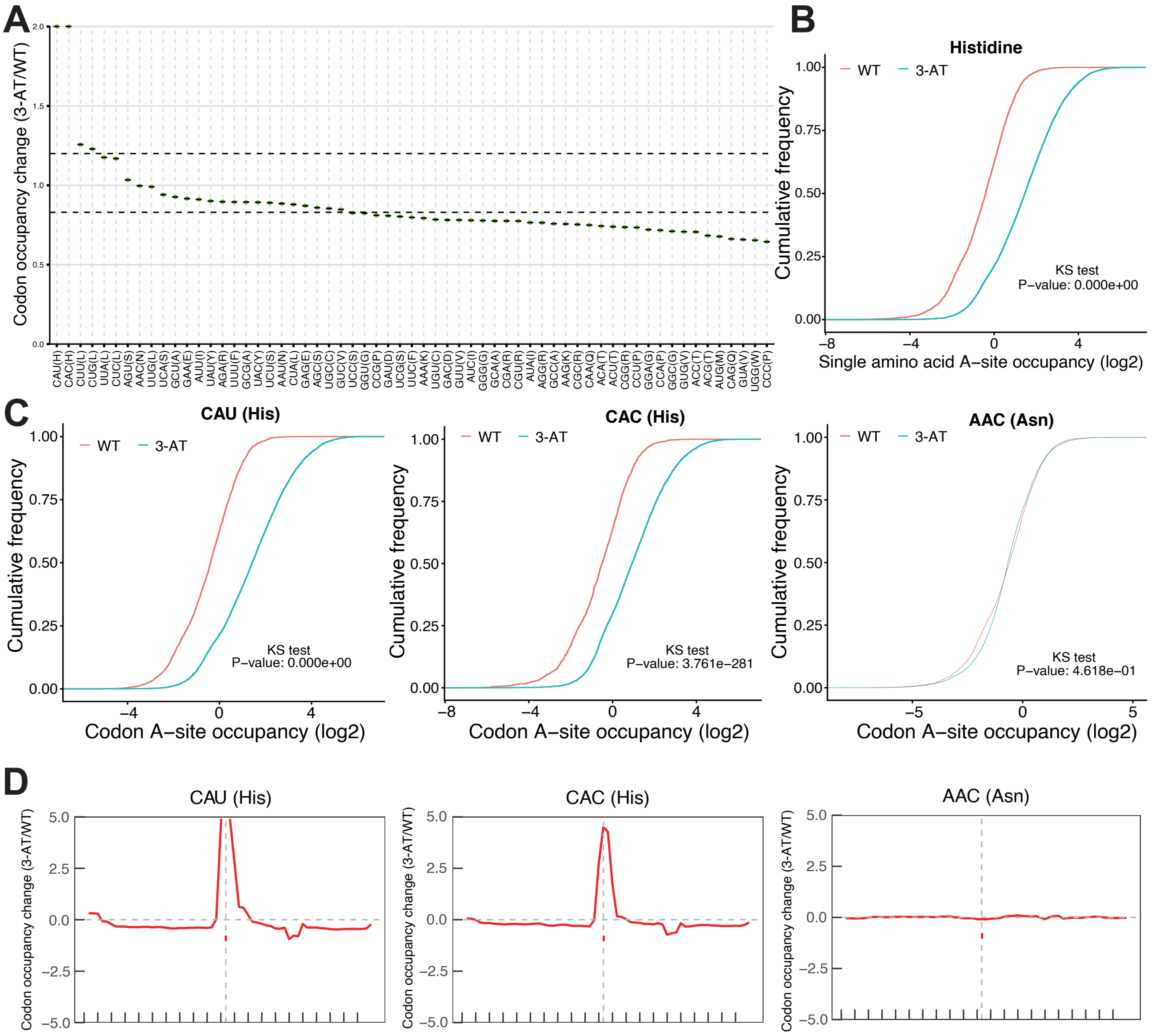


**Figure S1. RiboToolkit analyses showed significant Ribosome pausing at histidine codons in yeast treated with 3-amino-1,2,4-triazole (3-AT). (A)** Codon occupancy changes. **(B)** Cumulative occupancy distribution of histidine codons. **(C)** Cumulative occupancy distribution of individual histidine codons and AAC (Asparagine) is used as negative control. **(D)** Peaks of occupancy change in codon metagene plot.

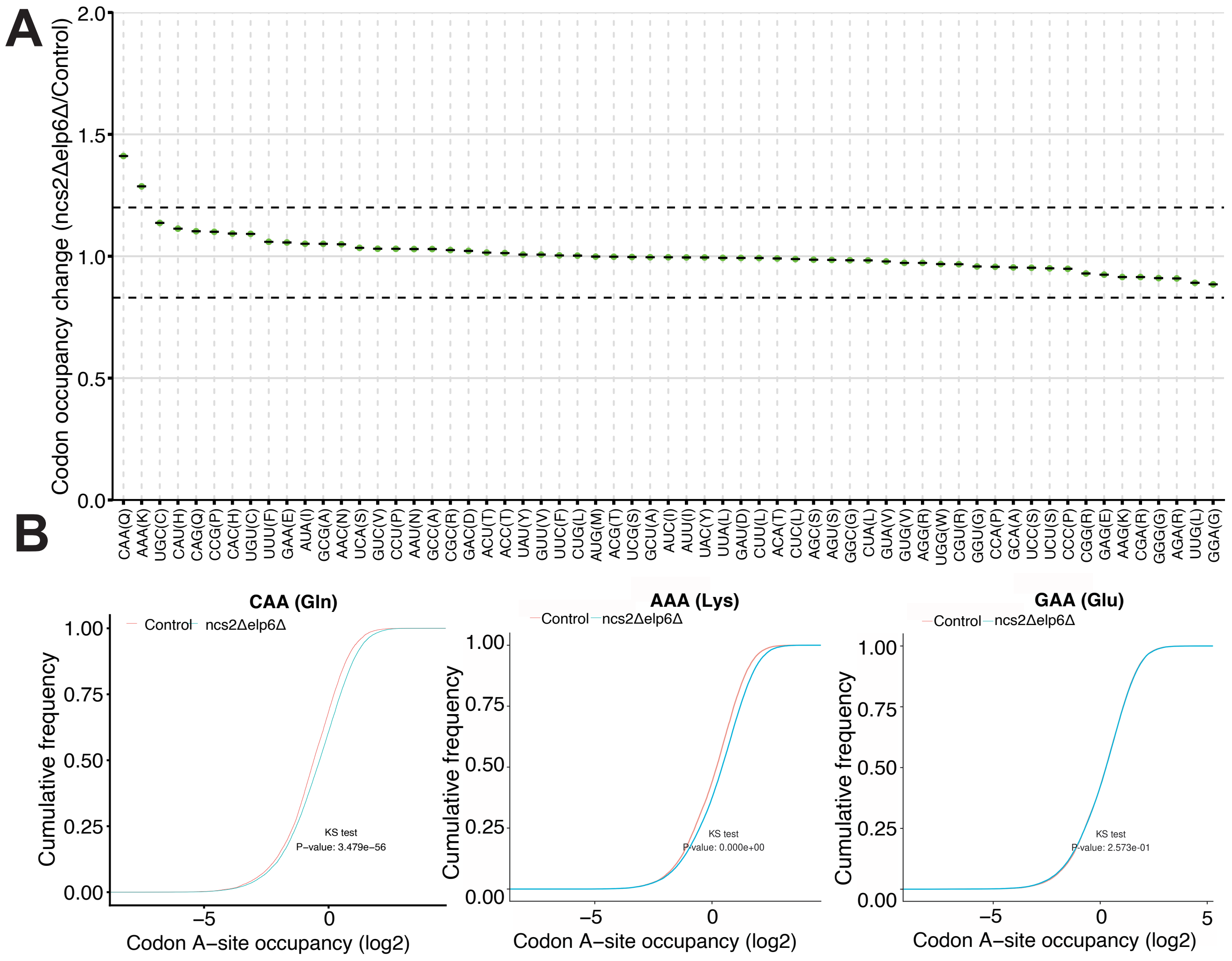


**Figure S2. Codon analyses by RiboToolkit of yeast ncs2Δelp6Δ mutant (lacking all U34 modifications) showed strikingly distinct effects of U34 modification loss on codon occupancy.** **(A)** Codon occupancy changes between yeast ncs2Δelp6Δ mutant and wide type; **(B)** Cumulative occupancy distribution of individual codons decoded by U34 modification tRNAs.

**Table S1.** Source of dataset used for the 16 supported model species

| | *Homo sapiens (hg19, hg38)* | *Mus musculus (mm10)* | *Rattus norvegicus (rn6)* | *Danio rerio (GRCz11)* | *Drosophila melanogaster (Dm6)* | *Caenorhabditis elegans (WBcel235)* | *Saccharomyces cerevisiae (R64)* | *Arabidopsis thaliana (TAIR10)* | *Oryza sativa (MSU7.0)* | *Zea mays (B73_RefGen_v4)* | *Glycine max (Wm82.a2)* | *Solanum lycopersicum (SL3.0)* | *Escherichia coli (k-12)* | *Bacillus subtilis (strain 168)* | *Pyrococcus furiosus (DSM 3638)* | *Halobacterium salinarum (NRC1)* |
|---|---|---|---|---|---|---|---|---|---|---|---|---|---|---|---|---|
| Reference genomes & mRNAs | | | | | | | | | | | | | | | | |
| GENCODE | √ | √ | | | | | | | | | | | | | | |
| Ensembl Genome | | | √ | √ | √ | √ | √ | √ | √ | √ | √ | √ | √ | √ | √ | √ |
| tRNAs | | | | | | | | | | | | | | | | |
| GtRNAdb | √ | √ | √ | √ | √ | √ | √ | √ | √ | √ | √ | | √ | √ | √ | |
| Ensembl ncRNAs | | | | | | | | | | | | √ | | | | √ |
| rRNAs | | | | | | | | | | | | | | | | |
| Ensembl ncRNAs | √ | √ | √ | √ | √ | √ | √ | √ | √ | √ | √ | √ | √ | √ | √ | √ |
| snRNAs | | | | | | | | | | | | | | | | |
| Ensembl ncRNAs | √ | √ | √ | √ | √ | √ | √ | √ | √ | √ | √ | √ | | | √ | √ |
| Functional gene annotations | | | | | | | | | | | | | | | | |
| Gene Ontology | √ | √ | √ | √ | √ | √ | √ | √ | √ | √ | √ | √ | | | | |
| KEGG | √ | √ | √ | √ | √ | √ | √ | √ | √ | √ | √ | √ | | | | |
| Reactome | √ | √ | √ | √ | √ | √ | | | | | | | | | | |
| Disease Ontology | √ | → | → | → | → | → | | | | | | | | | | |
| Network of Cancer Gene | √ | → | → | → | → | → | | | | | | | | | | |
| DisGeNET disease genes | √ | → | → | → | → | → | | | | | | | | | | |
| MSigDB | √ | | | | | | | | | | | | | | | |

The label "→" indicates that the data can be orthologically transferred to the relevant species from human.

**Table S2.** The feature comparison among integrated tools for ribosome profiling data analysis

| Features | RiboToolkit | RiboTools | riboSeqR | plastid | RiboProfiling | RiboGalaxy | Proteoformer | systemPipeR | RiboVIEW | riboStreamR | RiboFlow/RiboR/RiboPy |
|---|---|---|---|---|---|---|---|---|---|---|---|
| Software install type | | | | | | | | | | | |
| Web upload | √ | √ | | | | √ | | | | √ | |
| Batch uploading | √ | | | | | | | | | | |
| Local install | √ | | √ | √ | √ | √ | √ | √ | √ | | √ |
| Input types | | | | | | | | | | | |
| FASTA | √ | | | | | | | | | | |
| FASTQ | | | | | | | √ | √ | | | √ |
| SAM/BAM | | √ | √ | √ | √ | √ | | | √ | √ | |
| Table | | | | | | | | | | | |
| Link | √ | | | | | | | | | | |
| Quality control | | | | | | | | | | | |
| Contamination checking | √ | | | √ | | | √ | √ | √ | √ | √ |
| Quality checking | √ | √ | √ | √ | √ | √ | √ | √ | √ | √ | √ |
| RPF alignment | | | | | | | | | | | |
| RPF alignment | √ | | | | | | √ | √ | | | √ |
| Codon related analyses | | | | | | | | | | | |
| Codon occupancy | √ | √ | | √ | √ | √ | √ | | √ | | |
| Codon pause events | √ | | | | √ | | | | √ | | |
| Codon frequency | √ | | | | | | | | | | |
| Meta-codon plots | √ | | | | | | | | √ | | |

| | | | | | | | | | | | |
|---|---|---|---|---|---|---|---|---|---|---|---|
| Active ORF translation | | | | | | | | | | | |
| Active ORF calling | √ | | √ | | | | √ | √ | | | |
| Translation read through | | √ | | | | √ | | | | | |
| Proteomic analysis | | | | | | | √ | | | | |
| RPF expression and translational analyses | | | | | | | | | | | |
| mRNA expression | √ | | √ | √ | | √ | | √ | | √ | |
| RPF expression | √ | | √ | √ | √ | √ | √ | √ | | √ | |
| Translation efficiency analysis | √ | | √ | √ | | √ | | √ | | √ | |
| Differential translation analysis | √ | | √ | | | √ | | √ | | √ | |
| Functional analyses of differentially translated genes | | | | | | | | | | | |
| GO & KEGG | √ | | | | | | | √ | | | |
| Other pathways | √ | | | | | | | | | | |
| Gene Set Enrichment Analysis | √ | | | | | | | | | | |
| Other functionalities | | | | | | | | | | | |
| RPF distribution browse | √ | √ | | √ | | √ | | √ | √ | √ | √ |
| IGV mapping viewer | √ | | | | | √ | | | | | |
| RPF metagene plots | √ | √ | √ | √ | √ | √ | | √ | √ | √ | √ |
| Reproducibility between replicates | | | | | | | | | √ | | |

**Table S3**. Upload and analysis speed for different Ribo-seq datasets on the servers

| Data source | Sample IDs | Species | Number of Samples | Upload file format | Total file Size | Sever #1 | | | Sever #2 | | |
|---|---|---|---|---|---|---|---|---|---|---|---|
| | | | | | | Upload in the USA (Boston) | Upload in China (Chengdu) | Run time | Upload in The USA (Boston) | Upload in China (Chengdu) | Run time |
| GSE103667 | GSM2778762<br>GSM2778763<br>GSM2778764<br>GSM2778765 | Mouse | 4 | .fa.gz | 97 M | 1m36s | 1m57s | 25m | 3m3s | 2m45s | 13m |
| GSE67387* | GSM1646015<br>GSM1646016<br>GSM1646017<br><br>GSM1646024<br>GSM1646025<br>GSM1646026 | Yeast | 2 | .fa.gz | 289M | 5m12s | 5m42s | 49m | 8m23s | 6m57s | 26m |
| GSE52968 | GSM1279568<br>GSM1279579 | Yeast | 2 | .fa.gz | 67M | 1m5s | 1m12s | 16m | 1m58s | 1m36s | 8m |
| GSE112670 | GSM3076129<br>GSM3076131 | Mouse | 2 | .fa.gz | 44M | 42s | 50s | 21m | 1m15s | 1m6s | 11m |
| GSE32060 | GSM794854<br>GSM794855<br>GSM794856 | Mouse | 3 | .fa.gz | 78M | 1m6s | 1m18s | 18m | 2m36s | 2m12s | 8m |

“m” and “s” represent minute and second, respectively. “*” indicates three replicates are merged into one FASTA file to be uploaded to the server.
Server #1: https://rnabioinfor.tch.harvard.edu/RiboToolkit. Server #2: https://bioinformatics.sc.cn/RiboToolkit
